# Evaluation of Fibrin-Agarose Tissue-Like Hydrogels Biocompatibility for Tissue Engineering Applications

**DOI:** 10.3389/fbioe.2020.00596

**Published:** 2020-06-16

**Authors:** Fernando Campos, Ana Belen Bonhome-Espinosa, Jesús Chato-Astrain, David Sánchez-Porras, Óscar Darío García-García, Ramón Carmona, Modesto T. López-López, Miguel Alaminos, Víctor Carriel, Ismael A. Rodriguez

**Affiliations:** ^1^Department of Histology and Tissue Engineering Group, Faculty of Medicine, University of Granada, Granada, Spain; ^2^Instituto de Investigación Biosanitaria ibs.GRANADA, Granada, Spain; ^3^Department of Applied Physics, Faculty of Science, University of Granada, Granada, Spain; ^4^Department of Cell Biology, Faculty of Sciences, University of Granada, Granada, Spain; ^5^Department of Histology, Faculty of Dentistry, National University of Cordoba, Cordoba, Argentina

**Keywords:** fibrin-agarose hydrogels, *in vivo* biocompatibility, blood and biochemical profile, histological assessment, biodegradation, tissue engineering

## Abstract

Generation of biocompatible and biomimetic tissue-like biomaterials is crucial to ensure the success of engineered substitutes in tissue repair. Natural biomaterials able to mimic the structure and composition of native extracellular matrices typically show better results than synthetic biomaterials. The aim of this study was to perform an *in vivo* time-course biocompatibility analysis of fibrin-agarose tissue-like hydrogels at the histological, imagenological, hematological, and biochemical levels. Tissue-like hydrogels were produced by a controlled biofabrication process allowing the generation of biomechanically and structurally stable hydrogels. The hydrogels were implanted subcutaneously in 25 male Wistar rats and evaluated after 1, 5, 9, and 12 weeks of *in vivo* follow-up. At each period of time, animals were analyzed using magnetic resonance imaging (MRI), hematological analyses, and histology of the local area in which the biomaterials were implanted, along with major vital organs (liver, kidney, spleen, and regional lymph nodes). MRI results showed no local or distal alterations during the whole study period. Hematology and biochemistry showed some fluctuation in blood cells values and in some biochemical markers over the time. However, these parameters were progressively normalized in the framework of the homeostasis process. Histological, histochemical, and ultrastructural analyses showed that implantation of fibrin-agarose scaffolds was followed by a progressive process of cell invasion, synthesis of components of the extracellular matrix (mainly, collagen) and neovascularization. Implanted biomaterials were successfully biodegraded and biointegrated at 12 weeks without any associated histopathological alteration in the implanted zone or distal vital organs. In summary, our *in vivo* study suggests that fibrin-agarose tissue-like hydrogels could have potential clinical usefulness in engineering applications in terms of biosafety and biocompatibility.

## Introduction

The main objective of tissue engineering (TE) is to generate artificial biological substitutes to repair damaged human tissues and organs. Current tissue engineering protocols use different combinations of three basic components: cells, biocompatible and mechanically stable scaffolds and different bioactive factors to promote cell function and differentiation (Atala, 2012).

Scaffolds are essential tools in TE, since they define the biomechanical properties, physical dimensions, shape, and biological or physicochemical properties of bioengineered tissues (Campos et al., 2018). Among the numerous scaffolds used in TE, hydrogels have great potential due to their excellent biocompatibility due to their high hydration rate, diffusive and exchange properties allowing cell functions and viability (Ahmed et al., 2008; Carriel et al., 2014; Scionti et al., 2014). In this regard, one of the most widely used hydrogels in TE is fibrin, which offers some relevant advantages: low price, good cell-biomaterial interactions, fibrillary, and porous pattern and easy handling (Rosso et al., 2005; Swartz et al., 2005). Furthermore, fibrin hydrogels can be generated from the patient's own plasma and used in therapeutic protocols as an autologous product. Some of the bioartificial organs and tissues generated with fibrin hydrogels are skin (Meana et al., 1998; Helmedag et al., 2015; Keck et al., 2019), cornea (Alaminos et al., 2006), liver (Bruns et al., 2005; Wang and Liu, 2018), cardiovascular structures (Jockenhoevel et al., 2001; Mol et al., 2005; Myu Mai Ja et al., 2018), cartilage (Eyrich et al., 2007a,b; Almeida et al., 2016), and bone (Noori et al., 2017). Fibrin hydrogels have been used also as vehicles for delivering relevant products in wound regeneration (Banerjee et al., 2019). Although fibrin hydrogels have all the advantages indicated above, their biomechanical properties are typically poor as compared to the stiffness, flexibility, resistance, and strength of native tissues. In order to improve the biomechanical properties of fibrin hydrogels for tissue engineering applications, researchers combined this biomaterial with polyurethane (Lee et al., 2005), polycaprolactone-based polyurethane (Eyrich et al., 2007b; Wittmann et al., 2016) and polycaprolactone (Van Lieshout et al., 2006), among other biomaterials, with variable results.

Over the recent years, our group combined fibrin with agarose, a natural polysaccharide widely used in different laboratory applications and tissue engineering protocols (Alaminos et al., 2007). Fibrin-agarose tissue-like hydrogels (FATLH) allowed the successful biofabrication of different biological substitutes with promising *ex vivo* and *in vivo* results (Alaminos et al., 2006, 2007; Carriel et al., 2012, 2013, 2017a, 2019; Campos et al., 2016, 2018; Fernandez-Valades-Gamez et al., 2016; Rodriguez-Arco et al., 2016; Garcia-Martinez et al., 2017; Chato-Astrain et al., 2018). These studies demonstrated that the addition of agarose resulted in a significant improvement of the biomechanical properties as compared to fibrin hydrogels, especially when chemical crosslinkers were used (Campos et al., 2016, 2018). Potential clinical application of fibrin-agarose hydrogels are multiple, and include repair of damaged human organs such as the cornea (Alaminos et al., 2006), skin (Carriel et al., 2012), nerve (Carriel et al., 2013, 2017a; Chato-Astrain et al., 2018), oral mucosa (Garzon et al., 2013; Fernandez-Valades-Gamez et al., 2016), and cartilage (Garcia-Martinez et al., 2017), among others. In fact, our group has recently generated two of these models of bioartificial tissues that were approved as Advanced Therapies Medical Products (ATMP) by the National Medicines Agency in Spain (Agencia Española de Medicamentos y Productos Sanitarios—AEMPS) and are currently being used clinically in patients with and severe skin burns (Egea-Guerrero et al., 2019) and corneal ulcers (Gonzalez-Andrades et al., 2017; Rico-Sanchez et al., 2019). Although FATLH demonstrated very good results in preclinical studies in laboratory animals, a complete *in vivo* characterization of the mechanisms associated to FATLH biocompatibility is still needed.

Therefore, the aim of this study is to perform a time-course *in vivo* evaluation of the effects of FATLH grafted *in vivo* in laboratory animals to determine the stability of the graft over the time and the effect of the implant at the graft site (*in situ* response) and distal organs (liver, kidney, spleen, and lymph nodes). These studies were carried out at the histological (histochemical and ultrastructural) and laboratory levels (hematological and biochemical) as well as with magnetic resonance imaging (MRI).

## Materials and Methods

**Figure 1:**
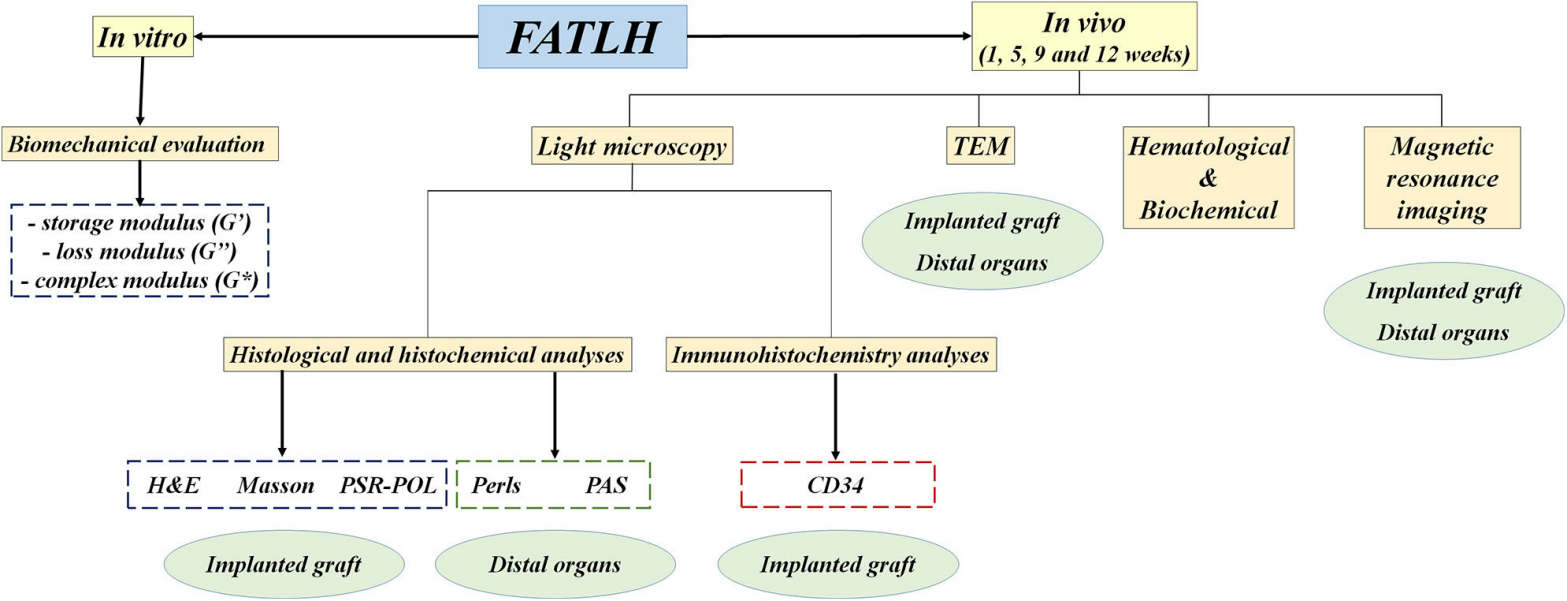
Description of the experimental plan developed in this work. FATLH were generated in the laboratory and analyzed *in vitro* and *in vivo* in laboratory animals as described in the materials and methods section.

A brief description of the experimental plan is sumarized in Figure 1.

### Generation of Fibrin-Agarose Tissue-Like Hydrogels (FATLH)

Fibrin-agarose tissue-like hydrogels (FATLH) were generated as previously described (Campos et al., 2016, 2018; Carriel et al., 2017a; Chato-Astrain et al., 2018). Briefly, to generate a FATLH with a volume of 10 ml, 7.6 ml of human plasma obtained from healthy blood donors were mixed with 750 μl of Dulbecco's modified Eagle's medium (DMEM) and 150 μl of tranexamic acid used as anti-fibrinolytic agent (Amchafibrin, Fides-Ecofarma, Valencia, Spain). Then, 500 μl of type VII agarose dissolved and melted in PBS to a final concentration of 2% were added to obtain a FATLH whose agarose content was 0.1%. Finally, 1 ml of a 1% CaCl_2_ solution was added in order to promote the fibrin polymerization reaction and the mixture was carefully mixed and aliquoted in Petri dishes with a diameter of 6 mm (5 ml per dish). This solution was allowed to solidify in a cell incubator at 37°C for 24 h.

### Biomechanical Evaluation of FATLH

FATLH were rheologically characterized to determine if these biomaterials are able to fulfill the basic biomechanical requirements of bioartificial tissues. The mechanical properties of FATLH were measured after 24 h of their preparation, with a controlled effort Haake MARS III rheometer (Thermo Fisher Scientific, USA). The measurement geometry used was parallel plates, which consisted of two disc with a diameter of 5 cm, where the surface in contact with the sample had roughness to prevent surface sliding. We performed two kinds of oscillatory experiments: amplitude sweeps and frequency sweeps. These measurements were used to calculate the storage modulus (*G*′), the loss modulus (*G*″), and the complex modulus (G^*^) as a function of shear strain frequency. In all cases, we maintained each frequency–amplitude pair during 5 oscillatory cycles, although we only used data for the last 3 cycles to rule out transients.

### Laboratory Animals, Surgical Procedures, and Experimental Groups

For this study, 12-week-old male Wistar rats weighing 250–300 g were used. These animals were maintained in the Experimental Unit of the University Hospital Virgen de las Nieves of Granada (Spain) and housed in a temperature-controlled room (21 ± 1°C) on a 12 h light/dark cycle with *ad libitum* access to tap water and standard rat chow. FATLH disc with 5 mm of diameter were subcutaneously implanted in the forelimb of each animal and evaluated after 1, 5, 9, and 12 weeks of follow-up (*n* = 5 in each time). Five healthy animals were used as controls (CTR) for all the analyses. Animals were euthanized by using anesthesia overdose and an infusion of a euthanasia agent followed by intracardiac perfusion of fixative. Experiments were performed according to the European Union and Spanish Government guidelines for the ethical care of animals (EU Directive No. 63/2010, RD 53/2013). Animal experimentation and the research work were approved by the local ethical committee (ref. 08-07-2019-121, AC17/013-NANOGSKIN).

### Histological and Histochemical Analyses

To carry out the *in situ* histological and histochemical evaluations of the grafting area—the subcutaneous tissue containing implanted FATLH—and distal organs, tissue samples from perfused animals were processed and embedded in paraffin as previously described (Carriel et al., 2017b; Chato-Astrain et al., 2018). Histological sections (5 μm thickness) were hydrated and stained with hematoxylin-eosin (H&E) for general histological assessment. Masson trichrome and picrosirius red histochemical methods were used to evaluate the presence and remodeling of the collagen network in each tissue type. These histochemical methods, especially picrosirius staining, are specific for the identification of fibrillar collagen fibers (types I, II, and III) due to the interaction of strong anionic dyes (sirius red F3B and light green) with the cationic groups of the collagen molecules (Kiernan, 2008; Carriel et al., 2011). Both methods are useful tools in diagnostic pathology and tissue engineering research (Wick, 2008; Chato-Astrain et al., 2018; Carriel et al., 2019). Images were taken using a Nikon Eclipse light microscope. At the same time, the Picrosirius Red Polarization Method (PSR-POL) method was used to identify and quantify the specific types of collagen fibers (mature fibers vs. recently synthetized fibers) as previously reported (Drifka et al., 2016; Chato-Astrain et al., 2020). Tissue sections stained with H&E and Masson trichrome were used to quantify the presence of specific cell types within the grafting site and in the grafted biomaterial using a morphological approach. In short, four expert histologists examined each tissue sample (controls and implants grafted for 1, 5, 9, and 12 weeks) and quantified the presence of local connective-tissue cells (i.e., fibroblasts and other cell types), pro-inflammatory cells (neutrophils and lymphocytes), and macrophages in a tissue area corresponding to 0.1 mm^2^. For each sample, we quantified 9 tissue areas inside the biomaterial and 9 tissue areas at the host grafting site. Tissue sections stained with H&E were also used to determine the biodegradation rate of the grated biomaterials. For this purpose, low magnification images were obtained from the implant site of each animal, which were analyzed using the Image J software (National Institute of Health, USA). In each case, the thickness of the implanted graft was quantified, and results were shown as percentage of biodegraded biomaterial as compared to the original graft implanted at time 0 used as a reference. A total of 10 measurements were obtained for each experimental group.

To evaluate the liver glycogen content and the basal membrane of renal glomeruli, the periodic acid-Schiff (PAS) histochemical method was used (Kiernan, 2008). To identify the presence of iron (Fe^+3^) in lymphatic node and spleen histological sections were stained with Perl's histochemical method (Perl's) contrasted with H&E as described previously (Rodriguez-Arco et al., 2016).

Finally, to identify the blood vessels found at the local implant site, we used immunohistochemistry with anti-CD34 primary antibodies (rabbit monoclonal anti-CD34 IgG, Abcam ab81289). Results were quantified to determine the percentage of area corresponding to CD34-positive cells by using the Image J software. Results were normalized using control tissues as a reference.

### Transmission Electron Microscopy (TEM)

For TEM, samples were fixed in 2.5% in cacodylate-buffered glutaraldehyde at 4°C for 4 h, rinsed in cacodylate buffer and then postfixed in 1% osmium tetroxide for 90 min. Then, samples were dehydrated and embedded in Epoxy resin (Carriel et al., 2012, 2017a; Chato-Astrain et al., 2018). Ultrathin sections were stained with aqueous uranyl acetate and lead citrate and examined with a transmission electron microscope (EM902; Carl Zeiss Meditec, Inc., Oberkochen, Germany).

### Analysis of Hematological and Biochemical Parameters in Blood

For analytical studies, blood samples were collected from each animal (*n* = 5 for each group and period) with 5% heparin to prevent coagulation (Chato-Astrain et al., 2020). The hematological profile was determined by using a Sysmex KX-21N automatic analyzer counter (Florida, USA) and the concentration of hemoglobin (HGB), red blood cells (RBC), hematocrit (HCT), platelets (PLT), white blood cells (WBC), lymphocytes (LYM), neutrophils (NEUT), and monocytes-basophils-eosinophils (MXD) was analyzed. In addition, the biochemical profile was determined in plasma by using a Cobas c311 automatic analyzer (Roche Laboratories, Basel, Switzerland). For biochemical tests, blood was centrifuged at 3,500 rpm during 15 min and afterwards the plasma was collected for the analysis of alanine aminotransferase (ALT), aspartate aminotransferase (AST), total bilirubin (BILT), direct bilirubin (BILD), urea (URE), and creatinine (CRE). All parameters were evaluated by using RTU kits from Roche Laboratories.

### Magnetic Resonance Imaging (MRI) Assessment

For MRI, animals were deeply anesthetized with isoflurane, placed in an MRI-compatible cradle, and scanned using a Biospec TM 70/20 USR instrument for laboratory animals (Bruker, Billerica, MA), equipped with 7 Tesla Ultrashield Refrigerated USR magnets. These analyses were performed at the animal facility of the Scientific Instrumentation Center of the University of Granada. First, a general scanning analysis was performed and high resolution axial T2-weighted datasets were acquired for the visualization of the whole animal. Second, we specifically analyzed the site of implantation and physiologically and immunologically important organs, such as satellite lymph nodes close to the grafting site, liver, kidneys and spleen (*n* = 3 in each time).

### Statistical Analyses

First, each variable was analyzed using the Shapiro-Wilk test of normality. As all variables resulted to be non-normally distributed, non-parametric tests were used to compare the results obtained among different study groups. To identify statistical differences between two specific groups of samples, the Mann-Whitney *U*-test was used. In the case of the percentage of biodegraded biomaterial, the exact test of Fisher was used, since values were expressed as percentages. All tests were carried out two-tailed using SPSS 16.00 software. Statistical *p*-values below 0.05 were considered as statistically significant.

## Results

### *In vitro* Rheological Characterization of FATLH

**Figure 2:**
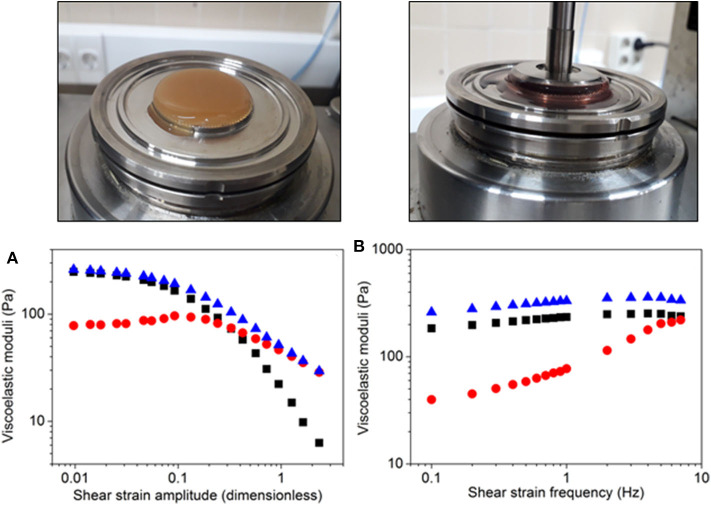
Rheological characterization of FATLH. Representative FATLH images are shown above, before and during the rheological analysis. **(A)** Viscoelastic moduli as a function of shear strain amplitude for imposed oscillatory shear strain of fixed frequency (1 Hz). **(B)** Viscoelastic moduli as a function of shear strain frequency for imposed oscillatory shear strain of fixed amplitude (0.02). Squares (▪) represent the storage modulus, circles (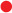) the loss modulus, and triangles (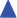), the complex modulus.

First, we obtained the viscoelastic moduli as a function of the amplitude of shear strain for a fixed frequency of 1 Hz. As observed in Figure 2A, the loss modulus, the storage modulus and the complex modulus showed approximately independent values of the shear strain amplitude at low values of this magnitude, corresponding to the linear viscoelastic region (LVR). As observed, within this region the storage modulus was much larger than the loss modulus (which is typical of crosslinked, gel-like systems) and, consequently, the complex modulus (which is defined as the root square of the sum of the squares of the loss modulus and the storage modulus) coincided approximately with the storage modulus. For larger values of the shear strain amplitude, the loss modulus begun to increase, whereas both the storage modulus and the complex modulus decreased. These changes marked the onset on the non-linear viscoelastic regime, for which the materials suffer irreversible changes at the microscopic level. As shown in Figure 2, the loss modulus exhibited a maximum around 0.1 of shear strain amplitude. This maximum is the so-called yield point, and corresponds to the onset of liquid-like flow of the material. Above the yield point, all moduli (loss, storage, and complex moduli) strongly decreased with shear strain amplitude. For larger values of the shear strain amplitude, we found a cross-over of the loss and storage moduli, for a shear strain amplitude value of ~0.2. Above this value, the loss modulus became progressively larger than the storage modulus, indicating a predominantly liquid-like behavior, due to the strong, irreversible breakage of the internal structure of the material. At the highest values of the shear strain amplitude, the storage modulus was negligible and, consequently, the complex modulus coincided with the loss modulus.

Afterwards, we obtained the dynamic frequency spectrum of the materials, at constant value of the amplitude of the shear strain within the VLR (Figure 2B). As observed, all moduli (loss, storage, and complex moduli) showed a tendency to increase with the frequency of shear strain. This tendency was more accentuated for the loss moduli, which was almost negligible at the lowest values of the strain frequency as compared to the storage modulus. However, it reached values comparable to these of the storage modulus at the highest frequencies. For the whole range of frequencies under study, the complex modulus appeared as appreciably larger than the storage modulus, as an indication of the relevance of both, storage of energy and loss of energy due to friction forces, under the applied oscillatory shears.

### *In vivo* Histological and Histochemical Analyses

**Figure 3:**
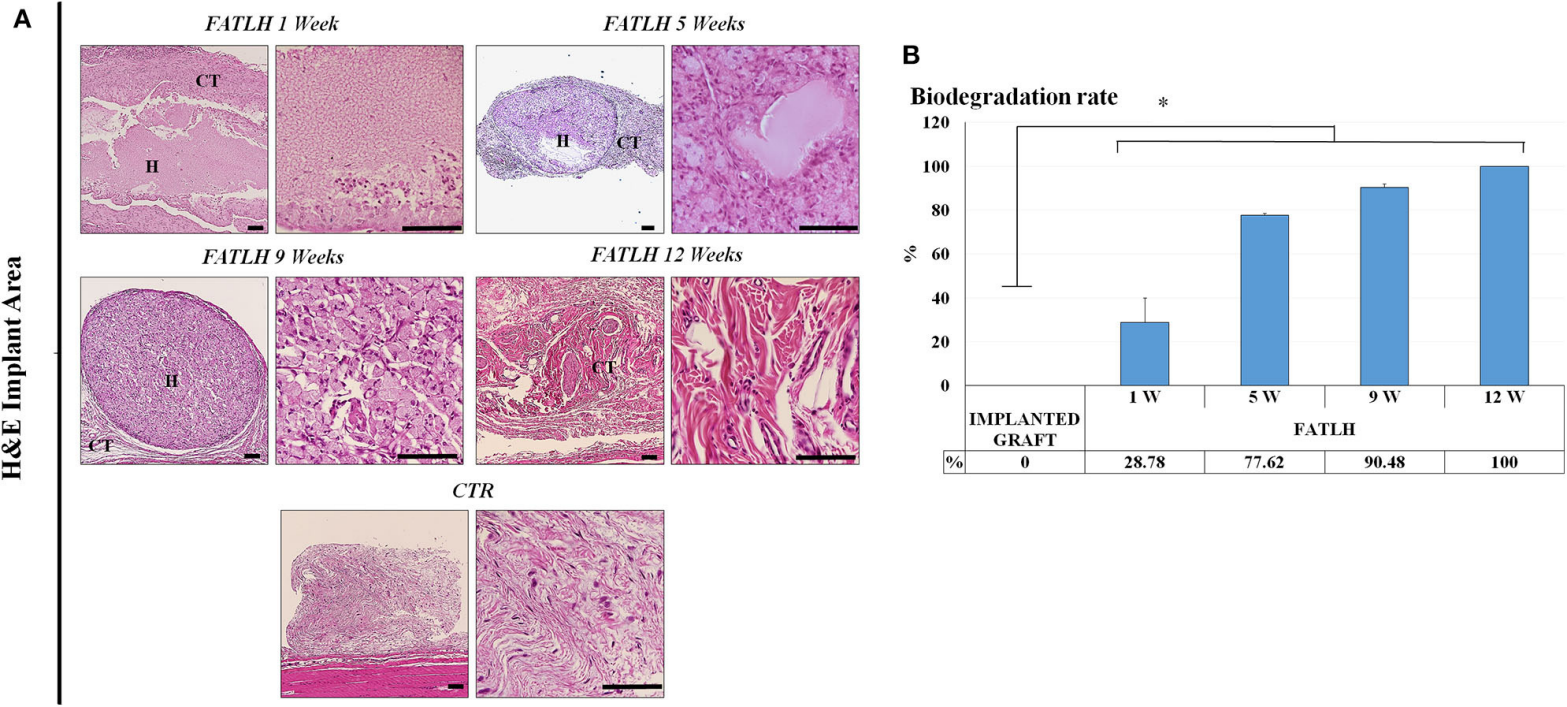
Histological analysis of FATLH grafted *in vivo* using hematoxylin-eosin (H&E). **(A)** Representative images of control samples (CTR) and the implanting site of the experimental groups (FATLH) at different follow-up times. H = grafted FATLH biomaterials; CT = connective tissue. Scale bar = 100 μm. **(B)** Quantification of the percentage of FATLH biodegraded at each study time (1, 5, 9, and 12 weeks). Results are shown as percentage of the implant that were biodegraded regarding the size od the original implanted graft. *Differences between each study time and the original size of the implanted graft were statistically significant.

**Figure 4:**
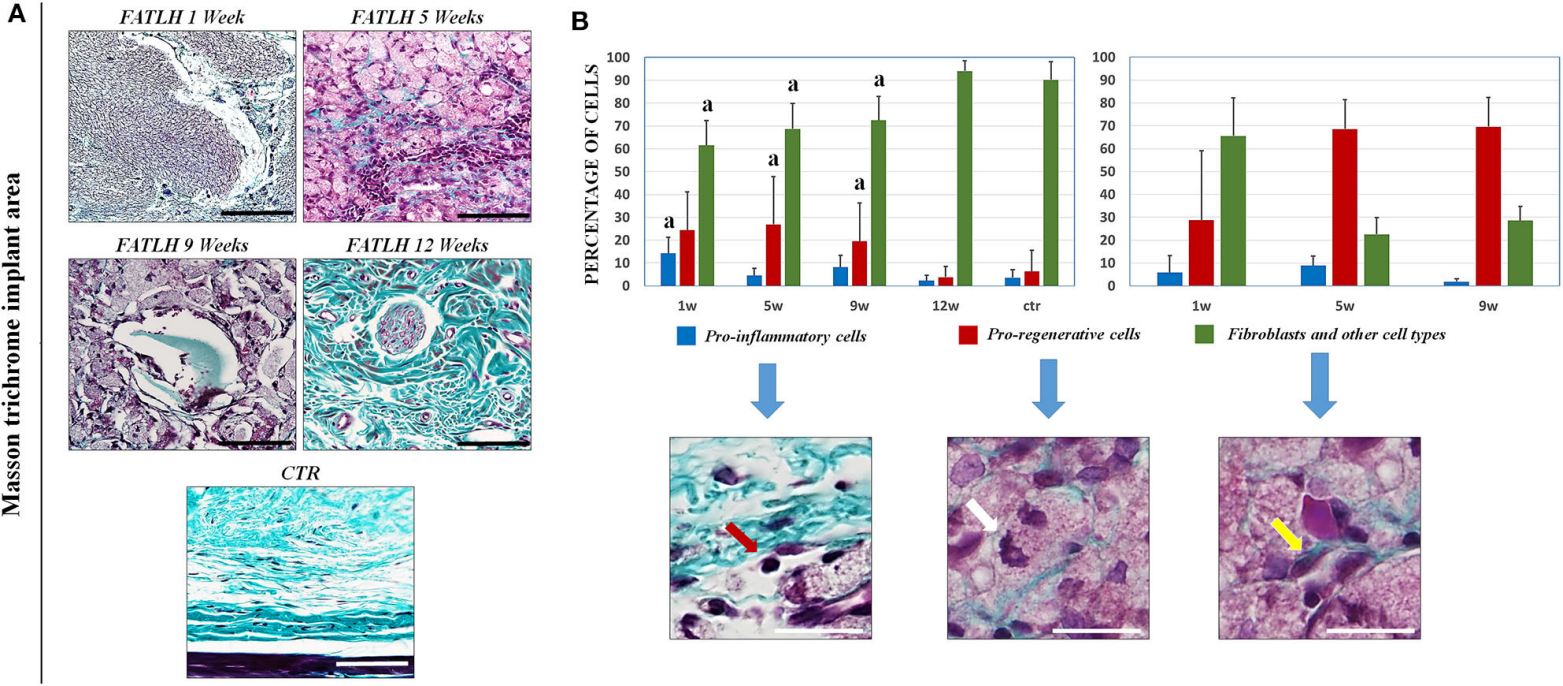
Histochemical analysis of FATLH grafted *in vivo* using the masson trichrome staining. **(A)** Representative images of controls and implanted FATLH at different periods of time. Scale bar = 100 μm. **(B)** Analysis of the types of cells found at the implant site (left panel) and inside the biomaterial (right panel). For the implant site, results are shown at 1, 5, 9, and 12 weeks of follow-up, whereas 1, 5, and 9 weeks samples are shown for cells colonizing the biomaterial, since controls and 12-weeks samples did not contain any grafted material. Results are shown as percentage of each cell type in each histological sample. “a” = significant differences vs. controls. Illustrative examples of each type of cells are shown and labeled with red arrows (pro-inflamatory cells), white arrows (pro-regenerative cells), and fibroblast and other type of cells (yellow arrows). Scale bar = 30 μm.

Histological analysis of FATLH grafted subcutaneously showed that the biomaterials tended to integrate with time in the host tissue, and no signs of necrosis, infection, hemorrhage, tumorigenesis, or other pathological processes were detected (Figure 3A). In controls and in all the study times, we found that the most abundant cells in the host tissue were the native connective cells, which mainly consisted of fibroblasts, endothelial cells, and other cell types (Figure 4). Regarding the pro-inflammatory host cells recruited to the graft site, we found an initial population of neutrophils and lympho-plasmocytic cells in samples corresponding to 1 week of follow-up. In these samples, differences with controls were statistically significant. In contrast, pro-inflammatory cells significantly decreased at 5, 9, and 12 weeks, with non-significant differences with controls at these times and a complete regression of the inflammatory reaction at 12 weeks. For the pro-regenerative cells, we found an abundant macrophage cell population in the area surrounding the implanted hydrogels, which corresponded to ~20% of the cells in the host tissue at 1, 5, and 9 weeks, and significantly decreased at 12 weeks, when values resembled those of controls. In the grafted biomaterial, we found that the most abundant cell population colonizing the graft were macrophages, especially after 5 and 9 weeks of follow-up. Host cells tended to form a capsule-like structure surrounding the biomaterial, with macrophages progressively driving the hydrogels biodegradation process from the surface inwards (Figure 4B).

Regarding the degradation rate of the grafted biomaterial, we found that the percentage of biomaterial that was removed from the implant site was 28.78% at 1 week, 77.62% at 5 weeks, and 90.48% at 9 weeks, with a complete biodegradation of the material at week 12 of the study (Figure 3B). Differences from each time of study vs. implanted graft at time 0 were statistically significant. Analysis of the grafted biomaterial revealed that FATLH were progressively colonized by host cells contributing to its biodegradation, mainly macrophages, although some fibroblasts were present especially at the first week (Figure 4B).

**Figure 5:**
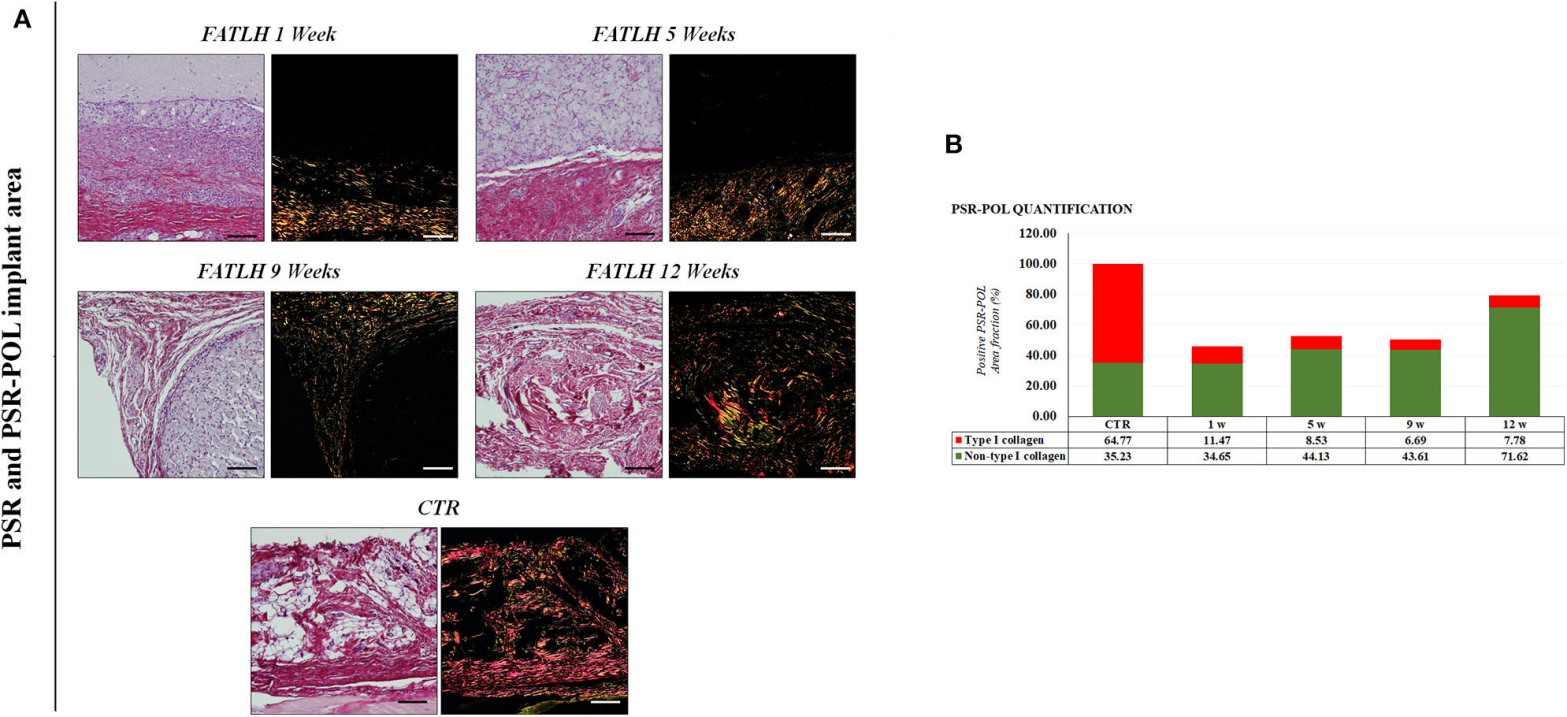
Histochemical analysis of FATLH grafted *in vivo* using Picrosirius red staining method with white and polarized light. **(A)** Analysis of controls and animals grafted with FATLH at different periods of time. Scale bar = 100 μm. **(B)** Quantification of different types of collagen fibrers using the PSR-POL method. For each sample, the total percentage of area occupied by type-I collagen (in red) and other collagen types (in green) is shown. Values are normalized regarding the controls.

When the ECM of the host tissue was analyzed, we found an active process of *in situ* synthesis and deposition of fibrillar collagen fibers as determined by Masson and Picrosirius red histochemical staining. As shown in Figures 4, 5, the synthesis of collagen fibers was progressive in the tissue surrounding the graft, resulting in a connective tissue similar to the control in 12-weeks samples. No signs of fibrotic response or other pathological processes were observed. Analysis using the PSR-POL method showed that control tissues were very rich in mature, thick collagen fibers stained in red that are compatible with type-I collagen (Zerbinati and Calligaro, 2018), with very few fibers stained in other colors. As shown in Figure 5 the amount of collagen synthetized at the graft site at 1, 5, and 9 weeks was lower than control samples, and fibers mostly showed orange, yellow, and green birefringence for the PSR-POL method, which could be considered as recently-synthetized non-type I collagen fibers (Zerbinati and Calligaro, 2018). At 12 weeks, the amount of collagen was higher than other weeks, although lower than controls, and no signs of fibrosis were detected (Figure 5). At 12 weeks, most of the fibers were orange, yellow, and green, as in previous study times.

**Figure 6:**
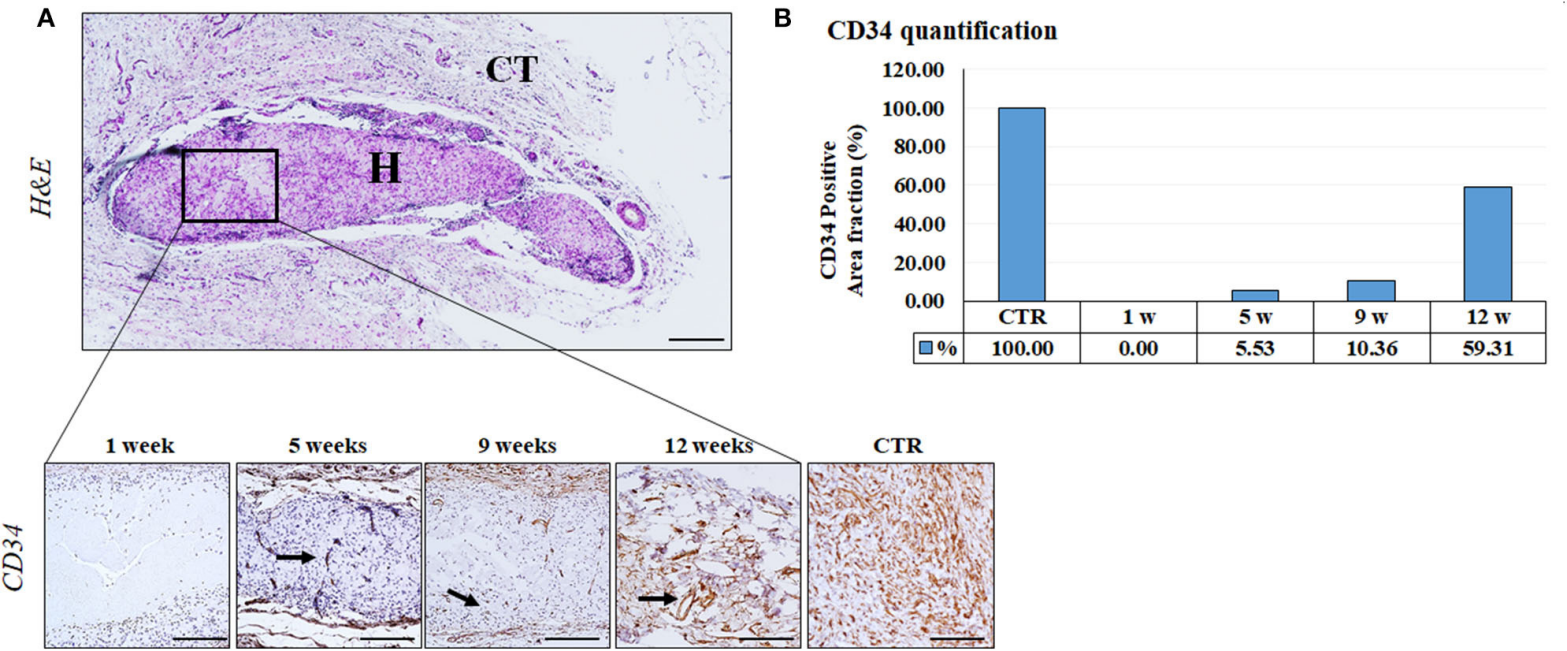
CD34 Immunohistochemical analysis of implanted FATLH over the time. **(A)** Low magnification section of the grafted biomaterial stained with H&E (top panel). H: FATLH, CT: surrounding connective tissue. Lower panel: Positive immunohistochemical reaction for CD34 (black arrows). Scale bar = 200 μm (H&E) and 100 μm (CD34). **(B)** Percentage of tissue surface occupied by CD34- positive blood vessels, normalized regarding the controls.

Furthermore, the immunohistochemical analysis and quantification of CD34 positive confirmed an active and progressive process of neovascularization inside the biomaterial, and newly-formed blood vessels were clearly identified within the grafted biomaterial from 5 weeks onwards (Figure 6). As shown in Figure 6B, the percentage of tissue occupied by blood vessels tended to increase with time.

**Figure 7:**
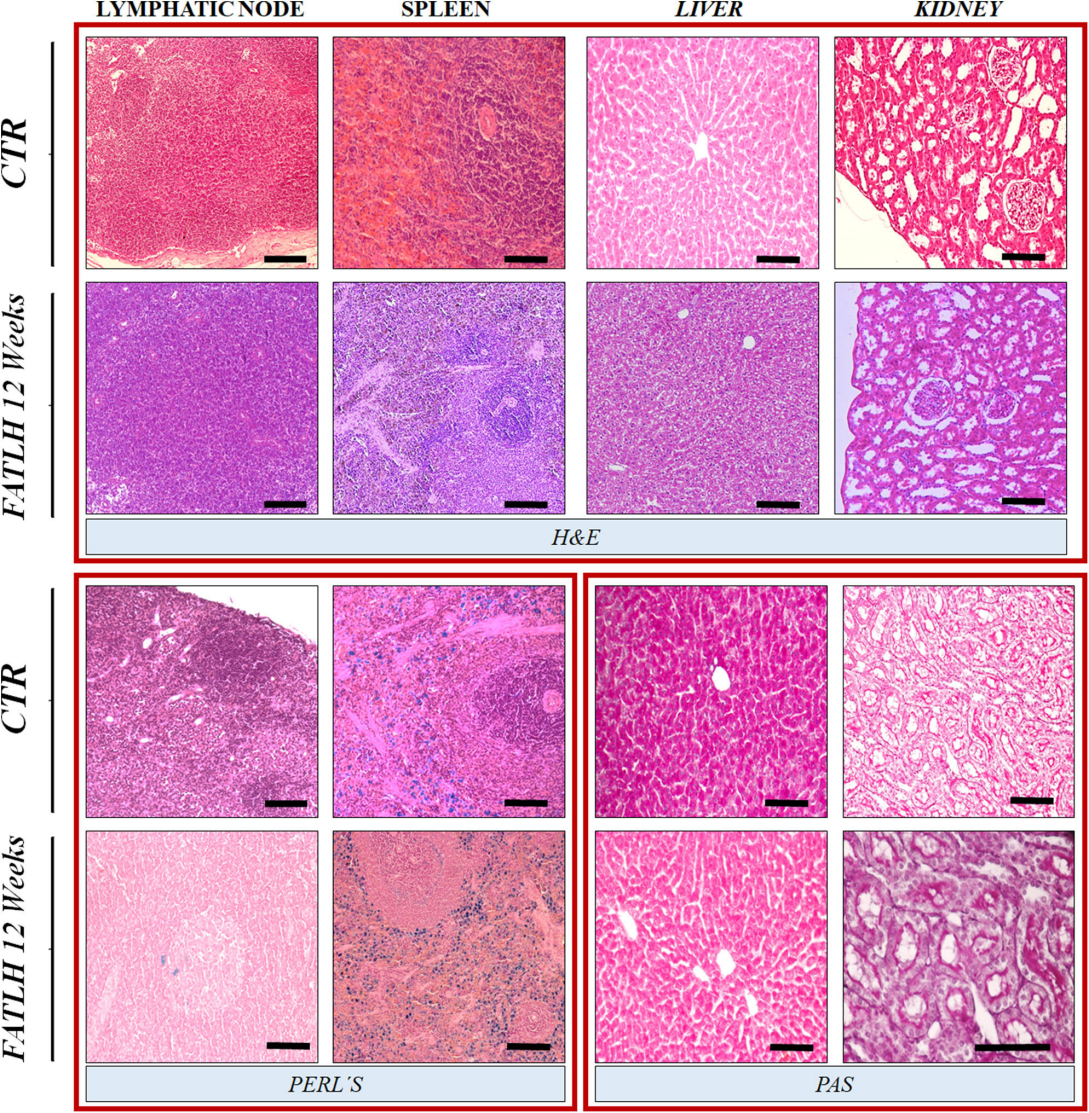
Histological and histochemical analyses of vital organs in controls and FATLH groups at 12 weeks. Representative images of the host lymphatic node, spleen, liver, and kidney are shown using hematoxylin-eosin staining (H&E), Perl's histochemical method (Perl's) and periodic acid of Schiff (PAS) staining. Scale bar = 100 μm.

The histological and histochemical analysis of the axillar regional lymph nodes did not revealed any pathologic signs of activation, edema or lymphadenopathy (Figure 7). Similarly, the histological evaluation of the spleen, liver, and kidney revealed a normal histological pattern and histochemical properties during the whole study as compare to the control (Figure 7).

### Ultrastructural Evaluation

**Figure 8:**
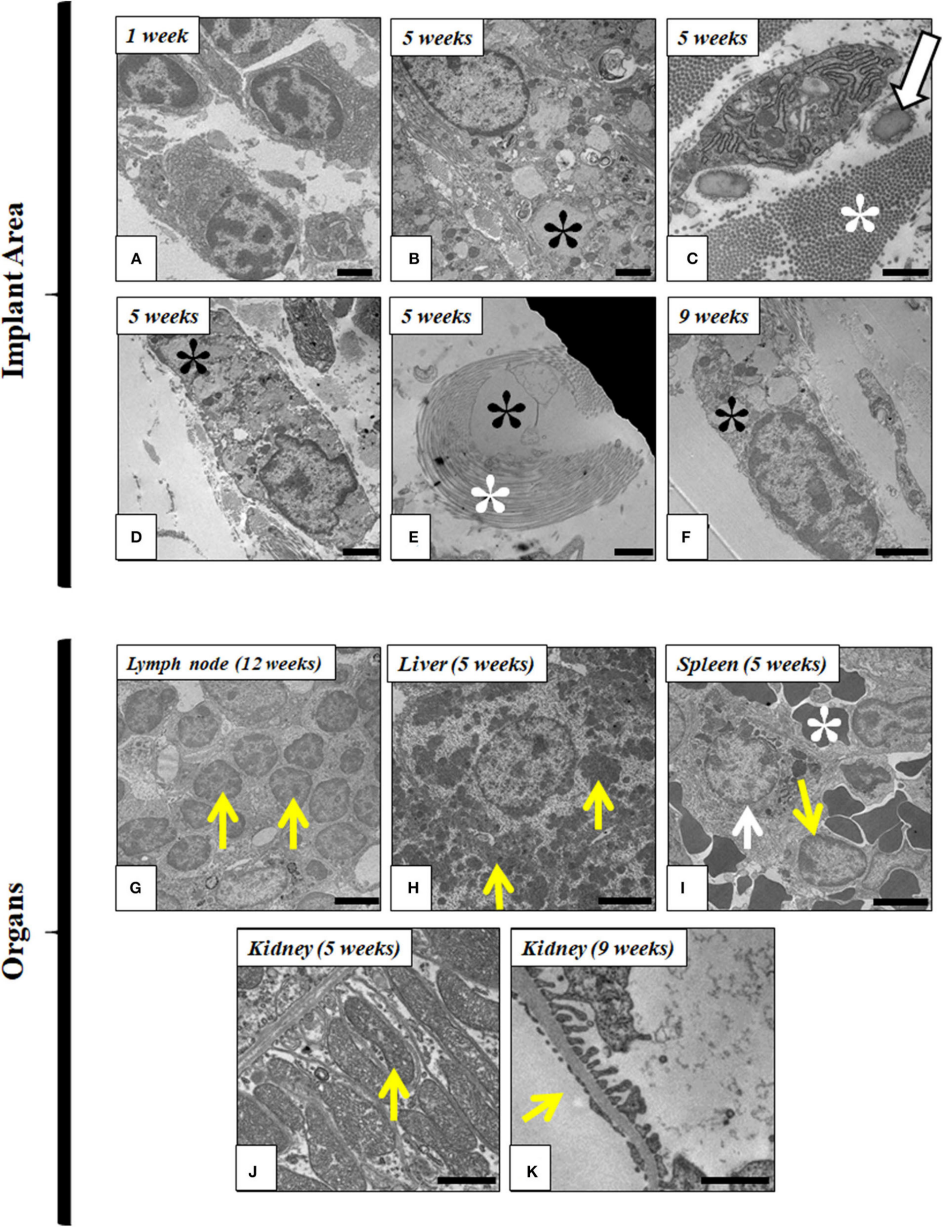
Ultrastructural analysis of FATLH implanted in laboratory animals. Representative images of the local implant site are shown in **(A–F)**, while distal organs are shown in **(G–K)**. **(A)** Plasmocyte cells at the implant site (1 week); Scale bar = 2 μm. **(B)** Macrophage phagocytizing fibrin-agarose (black asterisk*) (5 weeks); Scale bar = 2 μm; **(C)** Fibroblast cell immersed in a rich extracellular matrix containing collagen (white asterisk*) and elastic fibers (arrow) (5 weeks); Scale bar = 1 μm. **(D)** Macrophage phagocytizing fibrin-agarose (black asterisk*) (5 weeks); Scale bar = 1 μm. **(E)** Fibrin-agarose (black asterisk*) surrounded by collagen fibers (white asterisk*) (5 weeks); Scale bar = 1 μm. **(F)** Fibroblast phagocytizing fibrin-agarose (black asterisk*) (9 weeks); Scale bar = 2 μm. **(G)** Lymphocyte in satellite lymph node (yellow arrows) (12 weeks); Scale bar = 2 μm. **(H)** Hepatocytes containing glycogen (yellow arrows) (5 weeks); Scale bar = 5 μm. **(I)** Spleen macrophage phagocytizing iron (white arrow), lymphocyte (yellow arrow), and erythrocytes (white asterisk*) (5 weeks); Scale bar = 5 μm. **(J)** Basal region of proximal convoluted tubules of the kidney showing abundant mitochondria (yellow arrow) (5 weeks); Scale bar = 1 μm. **(K)** Glomerular filtration barrier in kidney (yellow arrow) (9 weeks); Scale bar = 1 μm.

Ultrastructural analyses were performed at the implantation site and distal organs. *In situ* analysis of the implantation site confirmed the presence of inflammatory cells, such as plasmocytes and lymphocytes, during the first week. In addition, a progressive decrease of these cells occurred over the time, and it was accompanied by an increase of macrophages tended at the grafting site. These macrophages showed clear signs of phagocytosis of the implanted FATLH until its complete biodegradation, which occurred between the ninth and twelfth week. Host fibroblasts also participated in tissue remodeling of the implanted area by generating collagen and elastic fibers that tended to surround the biomaterial. Interestingly, ultrastructural analysis revealed the presence of some fibroblasts with signs of phagocytosis within the grafted hydrogels (Figure 8).

The ultrastructural analysis of the vital organs did not reveal any modification of the normal histological pattern and/or cellular structure (such as the presence of inflammatory reaction, fibrosis, edema, cell necrosis, or dysfunction, etc.) confirming the results observed by the histological and histochemical assessment described above. In fact, normal cell components were observed in lymph nodes (mainly, lymphocytes), spleen (abundant cells and blood vessels), liver (hepatocytes containing glycogen), and kidney (normal ultrastructural organization of the ductal epithelial cells and the glomerular filtration barrier). Representative images are shown in Figure 8.

### Laboratory Testing and MRI

**Figure 9:**
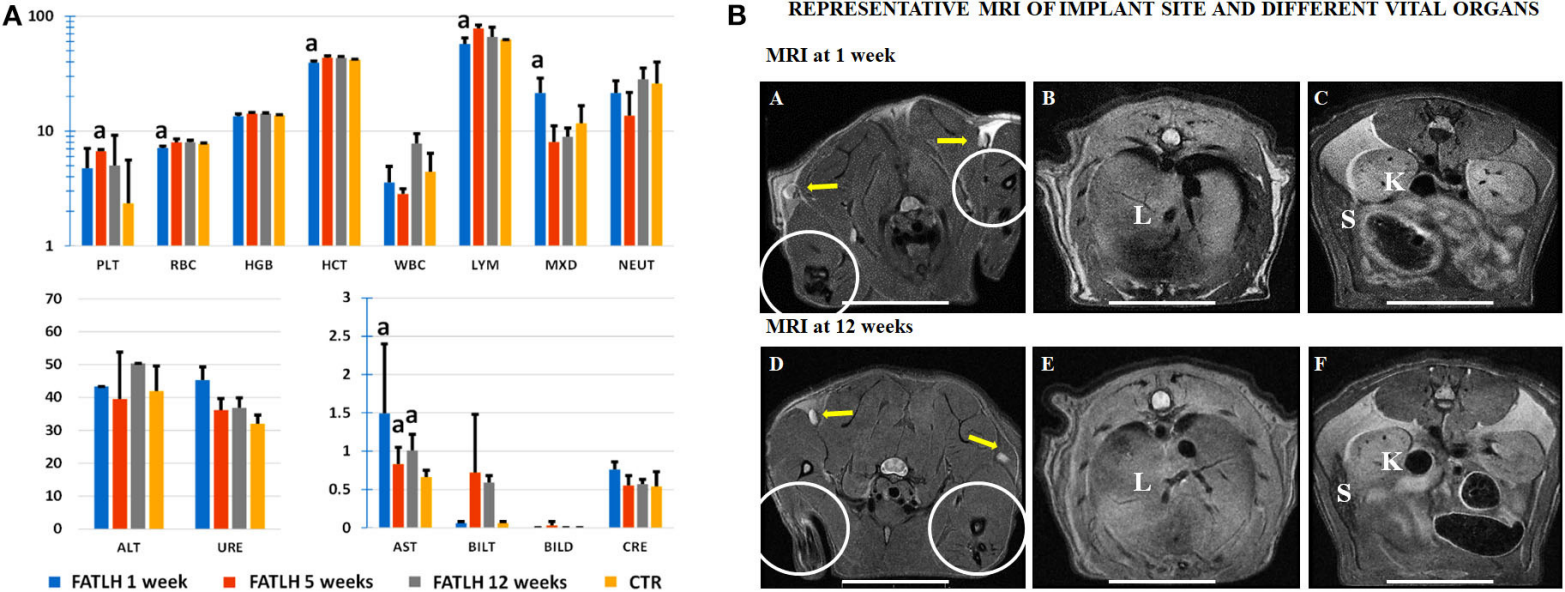
Laboratory testing and magnetic resonance images (MRI) analysis of controls (CTR) and animals grafted with FATLH. **(A)** Hemogram and biochemistry analysis in blood of control and study animals at different times (1, 5, and 12 weeks). PLT: Platelets (10^5^/μL^−1^), RBC: Erythrocytes count (10^6^/μL^−1^), HGB: concentration of hemoglobin (g/dL), HCT: hematocrit count (%), WBC: white blood cells (10^3^/μL^−1^), LYM: lymphocytes (%), MXD: monocytes-basophils-eosinophils (%), NEUT: neutrophils (%), ALT: Alanine aminotransferase (U/L), URE: urea (mg/dL), AST: aspartate aminotransferase (10^2^/U/L), BILT: total bilirubin (mg/dL), BILD: direct bilirubin (mg/dL), CRE: creatinine (mg/dL). Significant differences (*p* < 0.05) with the control group are highlighted with^a^. **(B)** MRI analysis of animals grafted with FATLH. Representative images of the implantation site and different vital organs are shown at 1 week and 12 weeks. The graft site is surrounded with circles; yellow arrows: satellite lymph nodes; L, liver; K, kidney; S, spleen. Scale bar: 2 cm.

In general, blood determinations in animals grafted with the FATLH showed some slight variations followed by a clear normalization of the evaluated parameters over time (Figure 9A). In this sense, grafted animals showed a significant reduction of the RBC count and HCT values after 1 week with respect to the CTR (*p* < 0.005). However, both parameters displayed a slightly increase and normalization at 5 and 12 weeks while the HGB concentration remained within the physiological range during the whole study (*p* > 0.05). Concerning the PLT values, they were higher than in the CTR group, but differences were statistically significant only in 5 weeks animals. In relation to the profile of the leukocytes, the WBC values in operated animals were comparable to the CTR group (*p* > 0.05), although an increase was observed at 12 weeks. When leukocytes were evaluated separately, we observed a significant decrease in the percentage of lymphocytes (LYM) after 1 week with respect to CTR (*p* = 0.02) and these values were normalized over time. Interestingly, the MXD (monocytes, eosinophils, and basophils) values showed a significant increase after 1 week (*p* = 0.02) as compared to the CTR group, but these values were clearly normalized at 5 and 12 weeks (*p* > 0.05). Finally, the neutrophils (NEUT) showed some slight variations over time, but these differences were not statistically significant (*p* > 0.05) as compared to the CTR group (Figure 9A).

On the other hand, the biochemical analyses carried out on the plasma of each animal showed a significant increase of the AST transaminase values after 1, 5, and 12 weeks (*p* < 0.05), while the rest of the parameters evaluated resulted comparable to the values observed in the CTR group (*p* > 0.05). Specifically, we found virtually no variations in the liver parameters ALT, total, and direct bilirubin (BILT and BILD, respectively) nor in the kidney markers creatinine (CRE) and urea (URE), as compared to control animals (Figure 9A).

The images obtained by MRI analysis of the grafting site and distal organs suggest that all analyzed tissues and organs were morphologically normal, and no alterations were detected using this method (Figure 9B). At the implantation site, no signs of inflammatory response, hemorrhage, edema, or local affection of the surrounding connective tissues and muscles were observed. When distal organs were evaluated (lungs, satellite nodes, pericardium and heart in thorax and digestive tract, spleen, kidneys, liver, pancreas, and mesentery in abdomen), we did not observe any sign of inflammatory process, presence of interstitial liquids, or fibrotic response. Indeed, the anatomical structure and dimensions of the thoracic and abdominal organs were found within the normal physiological parameters just like the CTR group (Figure 9B).

## Discussion

FATLH are gaining interest as scaffolds for the generation of human bioartificial tissues for the clinical treatment of patients with severe tissue damage (Gonzalez-Andrades et al., 2017; Egea-Guerrero et al., 2019). In previous studies, we evaluated the local tissue response after grafting several types of bioartificial tissues generated by tissue engineering containing different cell types and fibrin-agarose biomaterials, including, among others, the oral mucosa, skin, sclera, peripheral nerve, and palate (Carriel et al., 2012; Fernandez-Valades-Gamez et al., 2016; Garcia-Martinez et al., 2017; Chato-Astrain et al., 2018). Although these studies demonstrated the high biocompatibility and regenerative properties of FATLH (Carriel et al., 2019), a time-course biodegradation analysis and the impact of the implantation of these biomaterials on the structure and function of distal organs, as well as the global blood profile have not yet been determined. In the present study, we investigated the local effects of FATLH implanted in laboratory animals, along with the long-term potential impact on distal organs in order to assess and characterize the local and systemic effects of the *in vivo* implantation of FATLH.

First, our biomechanical analyses allowed us to confirm that the biomechanical behavior of FATLH was compatible with a clinical use. Firstly, the FATLH biomaterials of the present work exhibited values of the mechanical moduli within the VLR of about 200 Pa for the storage and complex modulus, and 80 Pa for the loss modulus. These values are similar to those reported in the literature for native tissues (Scionti et al., 2014), where values for several tissues were reviewed. For example, for native cornea and oral mucosa values in the range 40–100 Pa for the storage modulus and 5–40 Pa for the loss modulus were reported. Slightly higher values, in the range 400–2,000 Pa for the storage modulus and 100–600 Pa for the loss modulus, were reported for brain tissue and liver. Secondly, with respect to the tendencies of the viscoelastic moduli to increase with the frequency of the oscillatory shear strain, it should be noted that (Chan and Titze, 1999) reported similar trends for the storage modulus and the damping factor of native oral mucosa for the same range of frequencies of the present work. It should be noted that even though the loss modulus and the damping factor are different quantities, they both are related to the energy loss associated to the viscous response of a material, and therefore can be safely compared. From the point of view of materials science, the biomechanical behavior of FATLH represent a typical initial elastic behavior, followed by an irreversible alteration of the inner structure of the biomaterial as it is strained beyond its elastic limit. As a whole, the frequency spectrum showed by FATLH was similar to that of crosslinked polymeric systems (Rodriguez et al., 2013), and significantly higher than hydrogels consisting of fibrin alone (Egea-Guerrero et al., 2019). Although new methods able to improve the biomechanical properties of FATLH are in need, these results suggest that these scaffolds may be able to support the usual shear forces to which an artificial tissue is subjected once grafted *in vivo*. All these biomechanical values are compatible with a biomaterial with a high volume-based swelling degree (Q), as demonstrated by previous works (Bonhome-Espinosa et al., 2017). The high porosity of FATLH allows the incorporation of a high amount of molecules of water to generate a biomaterial with ~99.5% of water content (Scionti et al., 2014).

In the second place, we carried out a complete biocompatibility and biointegration analysis using a comprehensive approach including histological, hematological, biochemical, and imagenological approaches to support the clinical use of FATLH. The fact that all these methods coincided in the high degree of biocompatibility of the FATLH, they also confirm the safety of the *in vivo* use of these hydrogels in tissue engineering. In this regard, the use of highly-sensitive MRI image methods revealed that all animals were free from morphological alterations associated to the implant not only at the grafting site, but also in all major thoracic and abdominal organs. These results are in agreement with the histological and histochemical results obtained for distal organs, which were devoid of any alterations or modifications, suggesting that the subcutaneous implantation of FATLH is safe for the animals.

At the analytical level, we found that most parameters were normal in blood of the grafted animals, although some post-surgical changes were found. Thus, the transient decrease of RBC and HCT values and the transitory increase of the PLT count observed during this study could probably be related to the surgical procedure, which could generate this decrease that did not affect HGB concentration. The rapid recovering of all these values suggests that the FATLH is not able to generate any physiological alterations in the host animals at the hematopoiesis level. Similarly, some alterations were found for the percentage of LYM and MXD only during the first week, with a rapid normalization from the 5th week of the study. In general, this confirms the presence of a rapid, reversible post-surgical reaction in the host, which is likely associated to the surgical procedure rather than to the grafted material. The transient increase of the monocytes-basophils-eosinophils blood cell population may also be related to the subsequent increase of macrophages found at the grafting site, being these results supported by the well-known dynamics existing between circulating monocytes and tissue macrophages (Yona et al., 2013). Moreover, our results are in line with the normalization of the hematological profile observed in Wistar rats in which acellular nerve allografts were used (Chato-Astrain et al., 2020). All these results coincide with the normal profile of biochemical parameters found in plasma of grafted animals, except for AST. It is well-known that blood and biochemical parameters are often affected in individuals with different kinds of diseases, traumatic injuries, cancer, or organ rejection (Kampfmann et al., 2012; Barone et al., 2018; Putzu et al., 2018; Dasgupta et al., 2019). In our case, the increase found for the AST liver marker could be associated to the anesthetic procedure, since certain reports found that AST might be altered by the use of ketamine anesthetics, especially in young mice (Cheung et al., 2017). Similarly, Fang and cols. demonstrated a transaminase increase in pediatric patients sedated with ketamine (Fang et al., 2019). Whether or not the grafted FATLH may play a role in AST increase should be determined in future works. Apart from this transaminase, our results suggest that the FATLH graft was safe for liver and kidney functions. One interesting unsolved question is whether or not the biodegradation process of a fibrin-based biomaterial could affect the blood coagulation system. Although we did not find any signs of hemorrhage or other pathologies in tissues corresponding to the study groups, future studies should shed light on this issue.

At the histological level, we found that the local and distal host tissue reaction to the implanted hydrogels was compatible with a normal response to a local surgical procedure, and no significant alterations were found at the grafting site or distal organs. Analysis of the implanted biomaterials revealed a local reaction related to an active process of ECM remodeling with the synthesis of fibrillar collagen and elastic fibers. The density, organization, blood supply, and cellularity of the newly-formed ECM was compatible with normal connective tissue without any signs of fibrotic response against the implanted hydrogels, although the number of vessels was higher in control tissues than in FATLH groups. In fact, the amount of collagen fibers found at the different times was lower or comparable to controls, suggesting that a fibrotic process was not ongoing. Interestingly, the types of collagen fibers present at all time were generally thin, immature fibers that could be related to recently synthetized non-type I collagen fibers (Zerbinati and Calligaro, 2018), confirming the lack of a strong fibrosis process, which typically contain thick type-I collagen fibers (Rittié, 2017). This phenomenon is common in biocompatible bioartificial tissues grafted *in vivo*, and suggests that this type of bioengineered tissues may induce a physiological process of ECM synthesis and remodeling (Carriel et al., 2012, 2013, 2019; Fernandez-Valades-Gamez et al., 2016; Chato-Astrain et al., 2018, 2020). Future studies using primary antibodies should be carried out to identify specific collagen types in the different samples.

Regarding the mechanisms of *in vivo* remodeling and biointegration of the FATLH, we found that these biomaterials were actively biodegraded by host macrophages, which is in agreement with previous reports suggesting that these cells are the main responsible for material reabsorption (Aderem and Underhill, 1999; Parkar et al., 2009; Ratnayake et al., 2017; Chato-Astrain et al., 2018). It has been previously demonstrated that biomaterials grafted *in vivo* may induce two types of reactions in the host tissues: a pro-inflammatory or a pro-regenerative reaction. The first one is characterized by the presence of an inflammatory infiltrate mainly consisting of neutrophils, lymphocytes, and pro-inflammatory M1 macrophages. In contrast, pro-regenerative reactions mostly contain anti-inflammatory alternatively activated M2 macrophages associated to an active process of angiogenesis, ECM remodeling and tissue repair (Anderson et al., 2008; Jones, 2008; Al-Maawi et al., 2017; Sok et al., 2017). In this milieu, our histological analysis allowed us to demonstrate that the initial local inflammatory reaction found at the implant site was very limited and mainly consisted of neutrophils and lymphoid cells surrounding the graft, and this reaction decreased over time until complete resolution at 12 weeks. Importantly, the combination of fibrin and agarose did not generate a chronic foreign body inflammatory reaction characterized by the infiltration of abundant lymphocytes, plasmocytes, and giant multinucleated cells (Kamata et al., 2019). Instead, our analysis revealed that the predominant recruited cell types at all times were the macrophages, and tissues corresponding to 5, 9, and 12 weeks of development had very few pro-inflammatory cells and numerous macrophage cells. Although future studies are in need to determine if these macrophages had a M1 or M2 phenotype using specific cell markers, the active angiogenesis and ECM remodeling processes and the scarce number of pro-inflammatory cells found at the host tissues, lead us to hypothesize that the cell reaction driven by fibrin-agarose is pro-regenerative rather than pro-inflammatory. This would be in agreement with the lack of Langhans-type giant cells at the graft site, which are related to active inflammatory processes requiring macrophage fusion to allow biomaterial phagocytosis. The size of the particles resulting from the biodegradation of the biomaterials to be phagocytosed becomes a determining factor (Champion et al., 2008; Milde et al., 2015). Furthermore, our results are comparable with the host response that we observed in previous *in vivo* studies conducted with FATLH in rabbit models of scleral (Carriel et al., 2019) and palate repair (Fernandez-Valades-Gamez et al., 2016) in which the inflammatory reaction was very mild and self-limited and results were compatible with an active process of tissue regeneration and biomaterial integration. In addition, our analysis revealed the presence of an important population of fibroblasts both in the host tissue and colonizing the biomaterial. We observed that host fibroblasts were able to actively interact with the graft, migrate to the biomaterial and produce the ECM molecules that we found in our histochemical analyses, especially including collagens, and elastic fibers. Furthermore, the TEM analysis revealed that fibroblasts were able to migrate into the grafted hydrogels and contributed to its biodegradation by phagocytosis. Interestingly, it was recently demonstrated that, under certain circumstances, fibroblasts can acquire an active phagocytic phenotype, regulate inflammation, or even mediate angiogenesis (Shinde and Frangogiannis, 2017; Le Fournis et al., 2019), which would explain the results found in our study.

On the other hand, our results showed that complete biointegration and biodegradation of implanted FATLH was achieved between the 9^th^ and 12^th^ week of *in vivo* follow-up. Significant biodegradation coincided with a significant colonization of the biomaterial with macrophages and few lymphocytes and other pro-inflammatory cells. It is well-known that T lymphocytes may actively participate in the host response to grafted biomaterials (Anderson et al., 2008; Jones, 2008; Chato-Astrain et al., 2018) by mediating macrophage differentiation and the release of interleukins and interferons (Al-Maawi et al., 2017). As compared to previously published reports, the *in vivo* biodegradation process of FATLH was considerably faster than the 8 months observed for 1.5% agarose hydrogels implanted in Sprague-Dawley rats (Fernandez-Cossio et al., 2007), and slower than the ~3 weeks of fibrin hydrogels (Kawase et al., 2015). Based on our results, it is clear that the combination of fibrin with a low concentration (0.1%) of type-VII agarose is an efficient alternative to delay the *in vivo* biodegradation process of our FATLH up to 9–12 weeks without any unwanted local effects or impact on host cell viability. The incorporation of a low concentration of agarose in the FATLH did not affect the biological properties of the fibrin, but improved its biomechanical properties without the adverse effects often observed in high concentrations of agarose (Fernandez-Cossio et al., 2007; Ahmed et al., 2008; Zarrintaj et al., 2018). In fact, the host tissue became very similar to control connective tissue after the FATLH was completely degraded.

Biointegration is strictly dependent on the presence of a well-defined vascular supply and a proper capillary network generated by the host tissue. It is well-known that fibrin is a natural reservoir of numerous growth factors (Sanchez-Munoz et al., 2015) and pro-angiogenic molecules able to induce proliferation of host endothelial cells to create new blood vessels (Morin and Tranquillo, 2013). In contrast, it was demonstrated that agarose can only support a slight neovascularization process, and this could occur in about 4 months of *in vivo* implantation (Fernandez-Cossio et al., 2007). In our case, we found that grafted FATLH were able to promote an active neovascularization process in around 5 weeks, which is closely related to the time required for FATLH remodeling. These findings can partially explain the high degree of integration, neovascularization, and active tissue regeneration observed with our FATLH-based models of skin, nerve, sclera, and palate (Carriel et al., 2012, 2013, 2019; Fernandez-Valades-Gamez et al., 2016; Chato-Astrain et al., 2018; Egea-Guerrero et al., 2019; Martin-Piedra et al., 2019). In all these studies, the neovascularization was essential for the success of the tissue regeneration.

In summary, the present study demonstrated that the implanted hydrogels were progressively biodegraded, and biomaterials remnants were observed for up to 9 weeks, until the total integration and reabsorption. All these results suggest that FATLH could be used for transitory replacement of tissues and organs requiring up to 2 months of *in vivo* stability, such as the human skin, oral mucosa, palate, and cornea. These superficial organs are frequently damaged, and a temporal substitution with a bioengineered organ based on FATLH would allow and promote the replacement of the grafts by native host structures during this time period. In this regard, we previously found that these organs are typically remodeled *in situ* and replaced by native structure in a very short time (Alaminos et al., 2006; Carriel et al., 2012, 2013, 2019; Fernandez-Valades-Gamez et al., 2016; Chato-Astrain et al., 2018). Interestingly, peripheral nerve repair also require the use of temporal biomaterials able to promote neural regeneration by means of a complex process including axonal sprouting, ECM synthesis, and neoangiogenesis. This process takes around 2 months, depending on the length of the gap (Carriel et al., 2013; Chato-Astrain et al., 2018). In consequence, development of novel ATMP based on the FATLH described in the present work could contribute to the clinical treatment of several conditions requiring tissue regeneration within this temporal frame. Specifically, FATLH could be used for a regenerative repair of damaged corneas and skin burns, but also for the clinical substitution of other human tissues and organs such as the peripheral nerve (Carriel et al., 2013, 2017a; Chato-Astrain et al., 2018), oral mucosa (Garzon et al., 2013; Fernandez-Valades-Gamez et al., 2016) and cartilage (Garcia-Martinez et al., 2017).

One of the limitations of the present study is the lack of a control group in which an inert biomaterial is grafted subcutaneously. Although we used a control with no grafted material showing the biological repair reaction driven by the surgical procedure, an inert material would rather inform us about the reaction driven by the incorporation of a biomaterial with no biological function. Although previous experiments published by our group showed that inert polypropylene biomaterials grafted subcutaneously in the Wistar rat are not associated to a strong inflammatory or fibrosis reaction, and results were very similar to the controls used in the present study (Martin-Piedra et al., 2017), future studies should confirm these findings. Another limitation is the need of performing specific molecular analysis able to characterize the cells found at the graft site. These analyses would complement the detailed histological studies carried out in the study.

## Conclusions

In conclusion, this *in vivo* study confirm the high degree of biointegration and controlled biodegradability of FATLH, and suggests that these hydrogels could have potential clinical usefulness in engineering applications in terms of biosafety and biocompatibility.

## Data Availability Statement

The datasets generated for this study are available on request to the corresponding author.

## Ethics Statement

The animal study was reviewed and approved by Ethics and Animal Experimentation Committee of the University of Granada and the Andalusian Directorate of Agricultural Production (CEEA).

## Author Contributions

IR, FC, and VC designed the study, wrote the article, performed surgical procedures, analyzed histological and MRI results, and analyzed quantitative results. ML-L and AB-E performed rheological characterization. FC carried out the fabrication of cell-free fibrin-agarose tissue-like hydrogel. FC, JC-A, DS-P, and VC performed and analyzed the hematological and biochemical studies. FC, ÓG-G, and VC, carried out histological and histochemical studies. RC, IR, and VC performed ultrastructural studies. IR, MA, and VC performed a critical review and contributed to manuscript writing. All authors contributed to the article and approved the submitted version.

## Conflict of Interest

The authors declare that the research was conducted in the absence of any commercial or financial relationships that could be construed as a potential conflict of interest.
